# A Novel Wireless Wearable Volatile Organic Compound (VOC) Monitoring Device with Disposable Sensors

**DOI:** 10.3390/s16122060

**Published:** 2016-12-03

**Authors:** Yue Deng, Cheng Chen, Xiaojun Xian, Francis Tsow, Gaurav Verma, Rob McConnell, Scott Fruin, Nongjian Tao, Erica S. Forzani

**Affiliations:** 1 School for Engineering of Matter, Transport and Energy, Arizona State University, Tempe, AZ 85287, USA; ydeng29@asu.edu; 2 Center for Bioelectronics and Biosensors, Biodesign Institute, Arizona State University, Tempe, AZ 85287, USA; cchen99@asu.edu (C.C.); xiaojun.xian@asu.edu (X.X.); nongjian.tao@asu.edu (N.T.); 3 School of Electrical, Computer, and Energy Engineering, Arizona State University, Tempe, AZ 85287, USA; 4 Department of Preventive Medicine, University of Southern California School of Medicine, Los Angeles, CA 90089, USA; gverma@usc.edu (G.V.); fruin@usc.edu (S.F.)

**Keywords:** volatile organic compound (VOC), personal monitor, Photo Ionization Detector (PID), epidemiology study

## Abstract

A novel portable wireless volatile organic compound (VOC) monitoring device with disposable sensors is presented. The device is miniaturized, light, easy-to-use, and cost-effective. Different field tests have been carried out to identify the operational, analytical, and functional performance of the device and its sensors. The device was compared to a commercial photo-ionization detector, gas chromatography-mass spectrometry, and carbon monoxide detector. In addition, environmental operational conditions, such as barometric change, temperature change and wind conditions were also tested to evaluate the device performance. The multiple comparisons and tests indicate that the proposed VOC device is adequate to characterize personal exposure in many real-world scenarios and is applicable for personal daily use.

## 1. Introduction

Volatile organic compounds (VOCs) are organic chemicals emitted from anthropogenic and biogenic sources with significant vapor pressure [1]. Examples of VOCs are aromatic hydrocarbons, such as benzene, xylene, and alkanes such as hexane [2]. According to the United States Environmental Protection Agency (EPA), VOCs are among the major pollutants for indoor and outdoor air quality since they are emitted from furniture, appliances, and construction materials [3] as well as combustion of fuels (cars and trucks) [4]. Harmful VOCs typically are not acutely toxic, but have compounding long-term health effects [5]. Therefore, there is a need of determining real-time personal exposure concentrations. Many works have reported qualitative and quantitative analyses of indoor and outdoor VOC concentrations [6,7,8]. Popular methods such as photo-ionization detection [9] and gas chromatography (GC)-mass spectrometry (MS) [10] have been developed. In addition, other techniques such as thermal conductivity (TCD) [11], flame ionization (FID) [12], and differential mobility (DMD) [13] have been established to support detection with GC.

Quartz tuning forks (QTFs) are highly mass sensitive piezo-electronic resonators commonly used in watches for timing purposes and as biosensors for sensing applications [14]. By taking advantage of their small size and cheap cost, QTFs have been used as sensing elements for gas detection [15]. Our group has developed a wearable wireless monitor based on QTF sensors [16,17,18]. A film of molecularly imprinted polymer (MIP), which is selective and sensitive to target VOCs, has been applied on the surface of a QTF sensor. The MIP is a highly crosslinked polymer that is formed with a template molecule that is extracted afterwards, leaving selective cavities to create chemical affinity to molecules with similar structure [19]. When target VOCs molecules are adsorbed onto the polymer, a change in resonant frequency (induced from loaded mass change on the QTF sensor) is detected.

Compared to traditional detection methods, which are usually bulky or expensive, the QTF sensing mechanism gives the opportunity to develop non-intrusive, fast response, high sensitivity, and portable devices for personal VOC exposure detection. Studies in lab and field tests have been done to validate the specifications of a QTF-based VOC device [20]. The VOC device has shown the ability to provide timely response to VOCs exposure level, helping to identify potential health risks [21]. However, previous VOC device had an embedded QTF sensor inside the device and required special calibration protocols in the laboratory, which made it dependent on operator expertise. In addition, although the previous VOC device was relatively smaller compared to portable Photo Ionization Detectors, it was still too bulky to comfortably wear it for long periods of time. Furthermore, the VOC device required strict operation procedure to be followed, making operation less user-friendly.

In the present work, a new model of a VOC-sensing device based on QTF sensors is presented. Industrial, mass production, and design engineering work have been thoroughly performed to produce an end-user friendly VOC device as follows: (1) In order to avoid laboratory calibration procedures dependent on expertise, a replaceable pre-calibrated sensor cartridge has been created. (2) In order to improve wearability and encourage more user compliance (i.e., device wear-time), a two times smaller volume VOC device has been designed and mass-produced, including highly integrated components. (3) In order to produce an easy-to use device, a new user interface with embedded guidance has been created as a mobile application in a phone or tablet. It is worthy to mention that in many cases, a fully functional VOC-sensing device for real-world conditions is not trivial since stability of the materials, robustness of the sensor, and optimal device design must be met. Many lab-bench devices cannot reach this specific state and never become commercialized. In fact, there is a big gap between the number of publications in VOC-sensing devices published in the last 16 years (~34,000 publications) and the very few VOC monitors that are actually commercially available shows the difficulty of successfully implementing VOC-sensing devices in real applications. In order to validate the analytical, functional, and field-testing usability of the new VOC device presented in this publication, the performance of the VOC device is compared with existing commercial monitors such as a photo-ionization detector, gas chromatography-mass spectrometry, and carbon monoxide detector. In addition, environmental operational conditions, such as barometric pressure, temperature inversion, wind speed, and wind direction are also used to interrogate if the new VOC device’s performance is as expected. The multiple comparisons and tests indicate that the new wearable VOC device is suitable in characterizing personal exposure in real-world scenarios and is applicable for daily use. The progress reported here allows the present VOC device to reach future commercialization venues.

## 2. Materials and Methods

### 2.1. Device Sensing Mechanism

The personal VOC device based on QTF sensors consists of four functions: (1) Collection and delivery: the air sample is collected and delivered via an alternating channel valve-activated mechanism with a purging channel, delivering a VOC-free baseline for 120 s and a sampling channel, delivering the gas sample to analyze for 60 s. Environment hydrocarbons are tested through the sampling channel. Humidity of air sample is balanced by a Nafion^®^ tube. (2) Sensing and detection: after the air sample is produced, it passes a sensor chamber with a MIP-modified QTF sensor inside, and the sensing signal from the sampling vs. purging channels are compared and assessed. (3) Signal conversion and data transfer: the resonant frequency shift of the MIP-modified QTF sensor is the sensor signal output, which allows detection of gas phase analyte concentration due to the mass loading effect of the adsorption of molecules onto the MIP. (4) Data transmission and signal processing: the differential sensor signal is transmitted to a smart phone via Bluetooth^®^, where raw signal is captured, and further processed to provide concentration output. Detailed descriptions of the circuit have been provided in a previous publication [20].

### 2.2. Apparatus

#### 2.2.1. Photo-Ionization Detector (PID)

PID is one of the popular techniques for VOC concentration monitoring. A MiniRAE Lite photo-ionization detector from RAE Systems Inc. (San Jose, CA, USA) was used for comparison of VOC device performance, and real-time data from the PID monitor was recorded.

#### 2.2.2. Gas Chromatography/Mass Spectrometry (GC-MS)

Field tests at Mammoth Spring, Yellowstone National Park (WY, USA) were performed to validate the selectivity of the VOC device in an environment with high concentration of hydrogen sulfide, H_2_S. Since H_2_S does not belong to volatile organic compounds, although it is very common in some environmental scenarios such as petroleum refinery [22], H_2_S constitutes a major risk of interference for non-selective VOC monitors. To identify the existence of H_2_S, an air sample was collected and analyzed in a laboratory equipped with a GC-MS system (HP 5890 Gas Chromatograph interfaced to HP 5972 Mass Selective Detector Quadrupole Mass Spectrometer (Santa Clara, CA, USA) and with a Chromosil 330 column, Supelco, Sigma-Aldrich Co. (St. Louis, MO, USA). Prior to use, the GC column was conditioned overnight at 100 °C with dry carrier gas (nitrogen) at a flow of 20 mL/min. The gas sample was pre-concentrated in a polydimethylsiloxane coated solid phase micro extraction fiber (SPME) for 1 h, and desorbed from the SPME fiber into the GC at 200 °C. Selected Ion Monitoring mode was used in the mass spectrometry detector to get a better resolution of results and discrimination from interference. Identification of the analytes was performed using known standards and the mass spectrum library from NIST (AMDIS32 software, Gaithersburg, MD, USA).

#### 2.2.3. Carbon Monoxide Monitor

Carbon monoxide is very common in vehicle exhaust due to the incomplete combustion of gasoline [23]. It is also emitted to the environment along with VOCs, causing pollution. In order to test the correlation of the hydrocarbon concentration detected by the VOC device with carbon monoxide concentration, a commercial carbon monoxide (CO) (monitor Q-Track, Shoreview, MN, USA) was used to monitor carbon monoxide (CO) concentration to compare and correlate with the VOC device, and real-time data from the CO monitor was recorded.

### 2.3. Abbreviations Used Throughout This Work

In this paper, TVOC device stands for total VOC device and HC stands for hydrocarbon. The concentrations stated as part per million (ppm) refer to volume in volume concentrations (ppmV). For total VOC concentration, the unit ppmC indicates volume in volume total carbon concentration.

### 2.4. Field Testing’s Subjects

The Institutional Review Board (IRB) from Arizona State University # 1304009100 entitled A Pocket-Sized Device for Personal Exposure Level Assessment was followed. Four subjects wore and used the VOC device. None of them reported discomfort while using it. Furthermore, the subjects felt comfortable with the use of the monitor and found the user interface to be friendly.

## 3. Results and Discussion

### 3.1. Device Evaluation

#### 3.1.1. Device Development and Comparison

**Figure 1:**
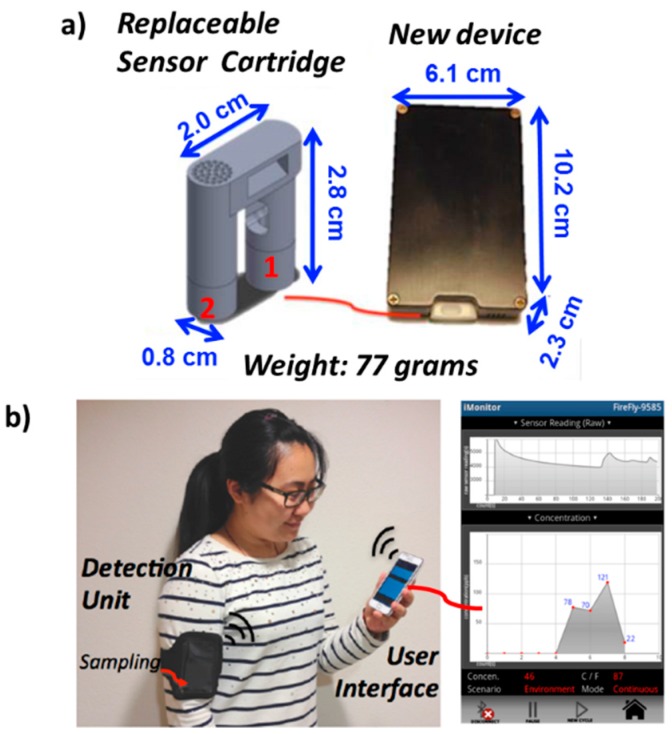
(**a**) Device dimension and weight; (**b**) Pictures of sensor components and user interface on a smart phone.

Figure 1a shows the new version of the VOC device, which is 2.1 times smaller in size than the previous generation device (12.7 cm × 7.6 cm × 3.6 cm). In addition, the new device is four times lighter in weight, making it a truly portable monitor, which is suitable to wear in a cell-phone type armband.

As mentioned before, another feature of the new VOC device generation is the replaceable pre-calibrated sensor cartridge for easy use of fresh and calibrated sensors during field studies. The replacement pre-calibrated sensor cartridges (part 1 shown in Figure 1a) are used with a QR-coded picture procedure, which is included in a guided protocol in the user interface of the device at the smart phone. In this way, former tedious sensor replacement (opening and closing of the housing), and in-situ gas sensor calibration is avoided by the replacement of the sensor element, and the use of a QR code containing the calibration factor of the “on-spot & ready-to-use” sensor. Each new replaceable sensor cartridge is also equipped with a chemical filter (part 2 shown in Figure 1a) for purging channel to ensure fresh filtering efficiency. The sensors have a lifetime of 464 ppmC∙h (see Table S1, and corresponding reference). A thermistor is also integrated in the flow channel to monitor temperature of the sensing environment, which is necessary for temperature correction of sensitivity (see Supplementary Materials). The QR code with pre-calibrated sensor information can guarantee the accuracy of tests for a period of 12+ h of continuous use (see Supplementary Materials). The decrease in size and weight of the device and introducing of replaceable sensor make the epidemiology tests with this device more convenient and user friendly (see below).

Figure 1b shows the components of the VOC device system, including the wearable VOC device and a smart phone with the user interface application installed. Figure 1b also shows plots of the real-time sensor signal (upper chart), and the environmental total hydrocarbon concentration (lower chart) in the user interface. The real-time sensor signal is captured every second and the environmental total hydrocarbon concentration is determined every three minutes. Detail specifications about this device are summarized in Table S1 in the Supplementary Materials.

#### 3.1.2. Device Calibration, Selectivity and Reproducibility of Sensors

**Figure 2:**
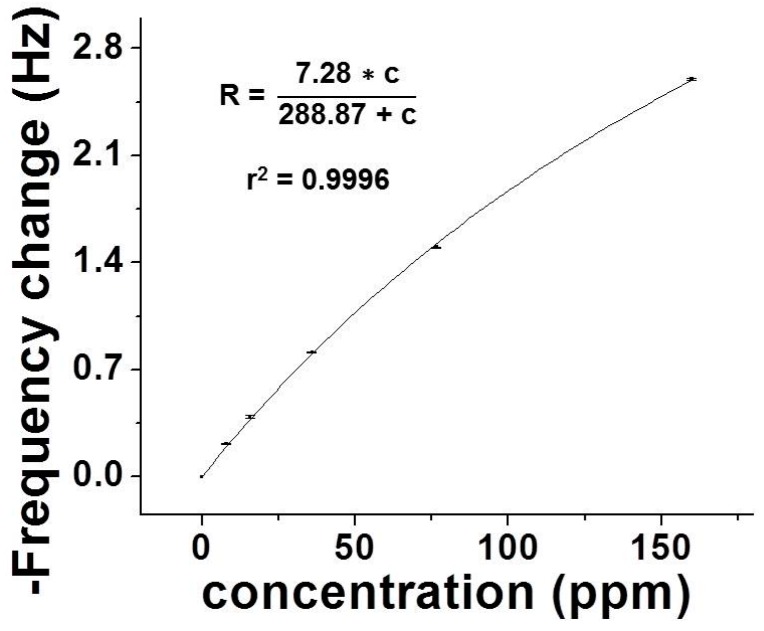
Sensor calibration under different concentrations of *o*-xylene.

Figure 2 shows a calibration curve of the new VOC device with a MIP-QTF sensor using the above-described setup. The calibration curve represents the sensor signal frequency change in presence of the sample vs. the absence of the sample (clean air baseline) under different concentrations of gas samples, including 0 ppm (clean air), 7.9 ppm, 15.8 ppm, 36 ppm, 76.5 ppm, and 160 ppm of *o*-xylene. *o*-xylene was chosen as model analyte to evaluate performance due to the abundance in traffic scenarios. The diluted samples were prepared in laminated bags with clean and dry air balance. All air sample concentrations were also monitored using PID as reference. The calibration curve is fitted to Langmuir adsorption isotherm, which is described by a relationship between the sensor response and analyte concentration with a fitting squared-regression coefficients (*r*^2^) close to 1.

As described in the Antoine equation [24], chemical vapor pressure rises with the increase in temperature. This will induce a temperature related difference on our sensor sensitivity since higher vapor pressure can reduce adsorption of the VOC molecule on the MIP. To ensure VOC device accuracy, we calibrated the VOC device’s sensor sensitivity under three different temperatures (see Figure S1a. Langmuir adsorption fitting was also applied for calibration. As discussed in our previous publication [19], the two calibration factors, R_max_ and K_D_, were defined in each fitted curve. The relationship between temperature and these two factors is presented in Figure S1b,c. It can be observed that as temperature increases, both R_max_ and K_D_ decrease. The relationship is considered to correct the VOC device’s sensor response to provide accurate analyte concentration readings. Further discussion of this temperature effect will be presented in future publications.

Hence we use calibration factors obtained from above (relationship between temperature with *R*_max_ and *K*_D_), Langmuir adsorption isotherm, and sensor manufacturing information to implement a QR code for each batch of sensor to achieve a better accuracy for point-of-use detection. A detailed description of generating this QR code is included in the Supplementary Materials.

It is important to mention that due to the different hydrocarbon distribution in different environmental conditions (i.e., indoor air, outdoor air, industrial air), different calibration factors have been defined for the VOC device in order to get the final calculated total hydrocarbon concentration in ppmC. These calibration factors are based on distributions of the different hydrocarbons, and the sensitivities of the MIP-QTF sensor to the individual hydrocarbons in the sample [20]. The hydrocarbon distributions were extracted from literature [25,26]. The different distribution scenarios can be chosen from the user interface of the new VOC device.

Selectivity of the MIP-QTF sensors to major hydrocarbons and interferents are shown in Figure S3. Another important feature of the new VOC device is the sensor fabrication reproducibility. MIP-QTF sensors prepared in batches of typically 200 sensors, fully assembled as replaceable sensors, and calibrated using artificial xylene sample showed a reproducibility of 9% (100% × SD/mean value).

#### 3.1.3. Personal VOCs Exposure Monitor

**Figure 3:**
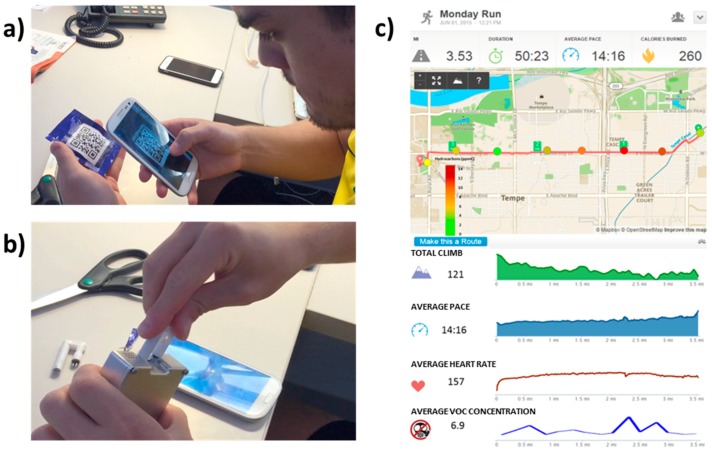
(**a**) Scanning sensor QR code; (**b**) Replacement of new sensor cartridge; (**c**) Real-time fitness and VOCs exposure device data during personal exercise.

To identify the effectiveness of real-time personal VOCs exposure monitor, the device was used to monitor the environmental air while a user was running from home to work on Arizona State University campus. Figure 3a presents the sensor QR code scanning procedure and Figure 3b shows the replacement of a new sensor cartridge before the test. The user wore the device on his armband during 50 min of running. Real-time results from the new total VOC device are shown in Figure 3c along with other fitness data (such as average pace and average heart rate) simultaneously recorded in the smart phone with a mobile application (Run Keeper^®^, Boston, MA, USA). The real-time VOC levels from the new VOC device are plotted and shown in comparison with the running route map. As shown in the map, when the user location approaches Arizona Highway Loop 101 and other major intersections, increases in VOC levels were observed due to the heavier traffic, which introduce more VOCs to the environment atmosphere.

This test demonstrates the ability of the device as a personal VOCs exposure monitor. With simple operation and an intuitive data presentation as shown in Figure 3, people can monitor their real-time VOC exposure level as simple as other fitness data.

### 3.2. Device Comparison with Current Technology and Commercial Device

#### 3.2.1. Response Comparison with Photo Ionization Detector (PID)

**Figure 4:**
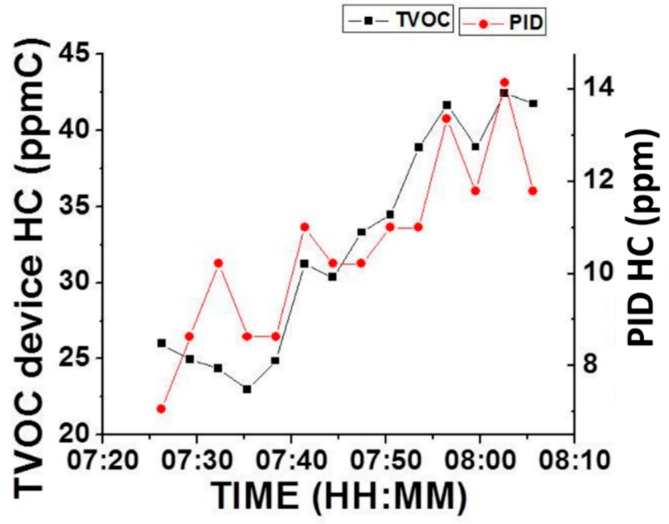
Comparison of the response between the new VOC (TVOC) device with a MIP-QTF sensor and RAE Photo-Ionization Detector (PID) for levels assessed during a trip on Los Angeles Highway 101. NOTE: The response of TVOC was calculated in ppmC using a calibration factor for “outdoor environment with motor vehicle exhaust” (see text for more details), while the response of PID was calculated in ppm using the calibration procedure described in the instrument manual.

The photo-ionization detector (PID) is a popular portable device for detecting volatile compounds by ionizing VOC molecules using a UV lamp [9]. In order to compare against current technology, a simultaneous test of the new VOC device with a PID was run with both devices placed inside a vehicle traveling along Los Angeles Highway 101 for ~40 min. The new VOC device gave a concentration value every three minutes. The PID monitor reading was also recorded at the same time. Figure 4 shows the response comparison between the new VOC device and the PID monitor. The scenario chosen to determine the total hydrocarbon concentration with the new VOC device was “outdoor environment with motor vehicle exhaust” distribution [20]. On the other hand, for the PID monitor, the correction factor for a mixture (CF_mix_) was calculated according to the PID manual, which indicates to use the sum of the mole fractions X_i_ of each component divided by their PID’s respective correction factors CF_i_ as follows:

CF_mix_ = 1/(X_1_/CF_1_ + X_2_/CF_2_ + X_3_/CF_3_ + ... X_i_/CF_i_) (1)

Due to the difference in data processing methods of the two monitors, the new VOC device gives hydrocarbon concentration in ppm Carbon (ppmC), while PID gives the concentration in ppm, rendering different final readings. However, as shown in Figure 4, overall average changes of readings from the two devices over time correlated well with each other (except for the first three points, where there is a potential confounding factor during device warm-up). A paired *t*-test between the relative values from the VOC device and PID was performed with *p* = 0.06 (see more details in Figure S4). This suggests that the new VOC device has comparable real-time effectiveness to detect changes of hydrocarbons as the PID monitor under an outdoor scenario with motor vehicle exhausts.

#### 3.2.2. Selectivity Validation Example with GC-MS

**Figure 5:**
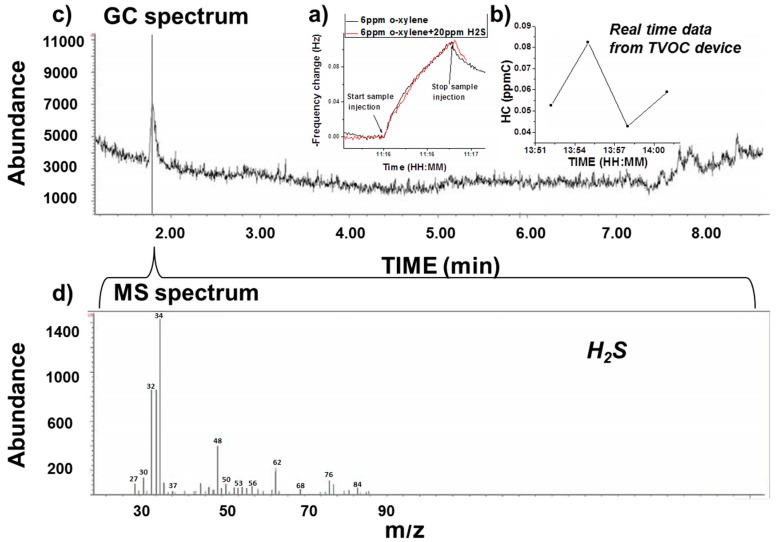
Selectivity validation test to H_2_S with artificial gas sample (**a**) and gas sample from Mammoth Spring, Yellowstone National Park. Real-time test was done on the new VOC device (**b**) and gas sample was collected for GC-MS analysis in the lab (**c**,**d**). The single peak in the chromatogram confirmed non-significant concentrations of other VOCs, and a presence of H_2_S.

Due to the nature of sensing material (MIP), MIP-QTF sensors are able to detect analytes in the hydrocarbon chemical family. In order to probe the selectivity of the sensors, an environment with a dominant single gas was chosen for field-testing, and the ambient air at Mammoth Spring, Yellowstone National Park was tested. Real-time concentrations with the new VOC device were assessed while air samples were collected for GC-MS analysis in the laboratory later. Figure 5 summarizes the results. Artificial gas sample was tested in the lab to show that the sensor can selectively respond to hydrocarbons in the presence of H_2_S. As shown in Figure 5a, response to 6 ppm *o*-xylene was recorded in the presence and absence of 20 ppm H_2_S. There was no significant change in response with the addition of H_2_S, confirming that the VOC device’s sensor is immune to H_2_S. Results of real-time hydrocarbon concentration from the new VOC device (Figure 5b) and analysis from GC-MS (Figure 5c,d) are compared. The hydrocarbon concentration given from the new VOC device was close to 0, indicating a low hydrocarbon concentration in the environment. However, the GC-MS analysis indicated the presence of a single strong peak of H_2_S (at 1.8 min in the gas spectrum). Although a quantitative analysis was not conducted (e.g., calibration curve on GC-MS), this qualitative result provides evidence of selectivity of the VOC device with MIP-QTF sensors in highly enriched H_2_S environments.

#### 3.2.3. Correlation with Carbon Monoxide Concentration

**Figure 6:**
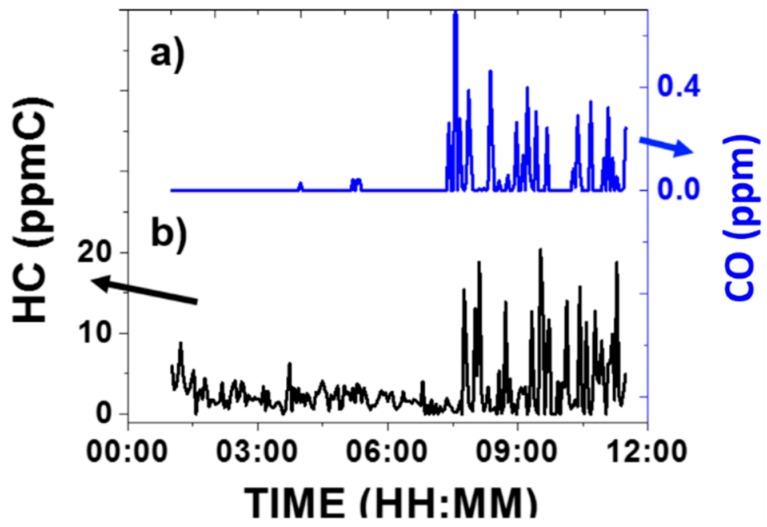
Outdoor testing of traffic markers as a function of time: (**a**) Concentration of Carbon Monoxide; (**b**) Corresponding concentration of total hydrocarbon (HC).

As mentioned above, a commercial carbon monoxide (CO) monitor was used to record CO concentrations while VOC concentrations were also recorded using the VOC device. The VOC device and the CO monitor were placed at the same spot, beside a local highway in Phoenix (AZ, USA) for 12 h. During this period, both hydrocarbon and CO concentrations were recorded continuously. Due to the difference in sensing mechanism between the devices, VOC device’s results were reported as an averaged concentration point every three minutes, while CO monitor gave an average concentration every minute. Figure 6 shows the real-time results for (a) CO concentration and (b) total hydrocarbon concentration profile, indicating that the two concentrations correlate very well, simultaneously showing: (1) A relative low level of target analytes due to light traffic from 00:00 a.m. to around 07:00 a.m.; (2) a relative larger fluctuating levels with higher concentration range from 07:00 a.m. to 12:00 p.m., coincidentally with heavier traffic. The temperature record indicated that there was no temperature inversion effect on the date of Figure 6. In addition, CO data was corrected by temperature. Furthermore, it is worthy to notice, that CO levels is expected to correlate with hydrocarbon levels, since both are incomplete combustion products of gasoline.

Same profiles could be reproduced in different days, reflecting a common profile of most daily transportation activities, which usually starts from morning and will stay busy during daytime. As mentioned in Section 3.1.2, since a big portion of HC concentration at residential comes from vehicle emission, the increase in traffic will cause a corresponding increase in HC concentration. The result demonstrated that hydrocarbon concentration from vehicle emission correlates well with real-time traffic pattern.

### 3.3. Device Validation under Different Scenarios

#### 3.3.1. Barometric Effect

**Figure 7:**
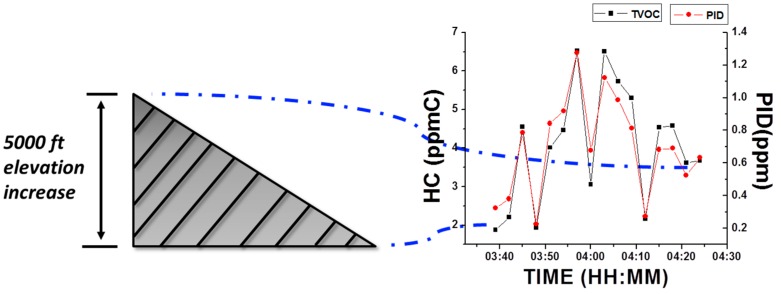
The new VOC device’s performance to a rapid elevation increase. Starting point and end point of the trip are indicated using dashed-dotted lines.

Pressure changes due to barometric changes are potential candidates that may affect device performance since it influences the environmental operational conditions of the tuning fork sensor, which can be sensitive to pressure [27,28]. In this field test, the VOC device was tested together with the PID monitor, while driving a car from the bottom of Trail Ridge Road, Rocky Mountain National Park (CO, USA) for 50 min, with an average speed of 35 miles per hour (MPH). This trip experienced a sharp elevation increase from 5280 ft to 12,183 ft, with an associated pressure drop of ~35 kPa from the beginning to the end [29]. As shown in Figure 7, both the new VOC device and PID monitor readings were recorded. It can be observed that the two devices were not affected by the barometric change in an obvious way, and the results were comparable with each other. This fact also demonstrated that the new VOC device can perform stably under sharp barometric changes. It is worth noticing that the immunity of the VOC device to pressure is attributed to the sample collection and delivery mode with a switching valve between purging and sampling channels, enabling baseline corrections right before each sensing period (as explained in the experimental section).

#### 3.3.2. Temperature Inversion Effect

**Figure 8:**
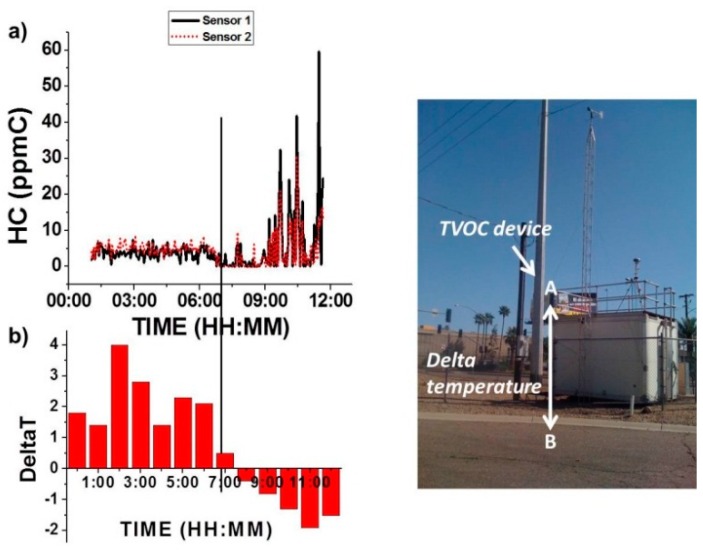
(**a**) VOC device’s real-time hydrocarbon concentration test at point A; (**b**) Corresponding delta temperature between points A and B. Height between point A and B was 14 ft.

A test was conducted to inspect the effect of ambient temperature change to the VOC device performance. As shown in the picture in Figure 8, the device (indicated as point A in the picture) was physically located on the roof of an environmental station. Hydrocarbon concentration was monitored for 12 h continuously together with the temperature from sensors located at points A and B. Figure 8 shows the HC concentration profile (a) in connection with the temperature profile (b) recorded for 12 h. From 00:00 a.m. to 07:00 a.m., the hydrocarbon level remained at a low level, reflecting a relative steady and low hydrocarbon concentration scenario. Starting from 07:00 a.m., HC concentration fluctuation increased, at which time, a change in temperature pattern was also noted (see below).

Figure 8b shows the temperature difference between point A (roof temperature) and point B (ground temperature) vs. time. A change of the temperature difference from positive to negative at ~7:00 a.m., known as inversion temperature, is shown in Figure 8b, and an obvious drop of HC concentration in the profile at the same time (~7:00 a.m.) is shown in Figure 8a. When the difference between roof temperature and ground temperature is changing from positive to negative, a well-known natural convection phenomenon occurs [30], leading to a dilution of pollutants in the air. This is expected to reduce the concentration of hydrocarbon, and was reflected as a transitory decay of HC concentration reading from the VOC device. This result shows the effectiveness of the VOC device to capture real-time HC concentrations due to environmental changing conditions such as temperature.

#### 3.3.3. Wind Condition Effect

**Figure 9:**
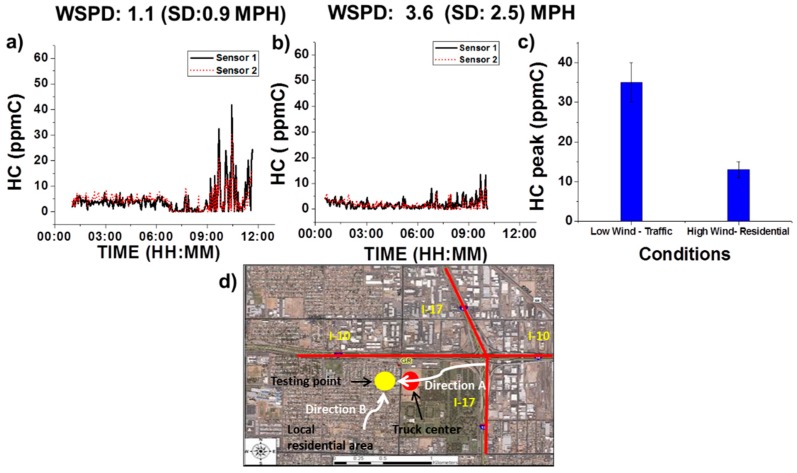
Wind speed and direction effect on VOC device performance. (**a**) HC concentration under lower wind speed and wind direction from areas with higher expected concentration (direction A showing in (**d**), (**b**) HC concentration under higher wind speed and wind direction from areas with lower expected concentration (direction B showing in (**d**); (**c**) peak HC values comparison; (**d**) a map showing the testing location and wind directions.

Wind condition will cause gas movement and redistribution of atmospheric components. To study the wind effect, we chose an environmental station from Maricopa Air Quality Department at the intersection of Arizona highways I-10 and I-17. The VOC device was placed at the same spot for two business days, for 12 h each day, detecting the hydrocarbon (HC) concentration profile. Wind speed and direction were recorded simultaneously. The data obtained from two testing days are presented in Figure 9a,b. Average wind speed on Day 1 was (1.1 ± 0.9) MPH and direction was from the east side of the testing location, which is marked as Direction A in Figure 9d. Average wind speed on Day 2 was (3.6 ± 2.5) MPH. Direction B in Figure 9d indicates the wind direction on Day 2. Direction A was from Arizona I-17 Highway and through a local truck center, which might potentially bring VOCs towards the testing location. Direction B was from a local residential area, which usually has low VOCs concentration.

Similar to the cases described in Section 3.2.3 and Section 3.3.1, both Figure 9a,b shows a relatively low HC concentration from 00:00 a.m. to 07:00 a.m. due to the light traffic and low activity level. However, a significant difference is observed in the time window from 07:00 a.m. to 10:00 a.m., with the case shown in Figure 9a having a much higher level of HC concentration than the case shown in Figure 9b. Since the two testing days were normal business days, traffic conditions on each day were expected to be similar. However, the HC concentration profiles (Figure 9a,b) and peak HC concentration values (Figure 9c) on the two days were significantly different. This result suggests that higher HC concentration levels were associated with lower wind speed and wind direction from areas with higher traffic such as a local truck center and highway. Likewise, higher wind speed and wind direction from cleaner locations had a lower expected HC concentration.

It is worthy to mention that in our sensor cartridge design, the small size of the sensor allows the assembly of the two identical sensors (sensors 1 and 2) inside the same sensor cartridge. The similar profiles (shown in Figure 9a,b) by the two sensors suggested reproducible environmental hydrocarbon detection capability of the VOC device. In addition, the dynamic response of the sensors in real-time remains constant throughout the lifetime of the sensors is shown in Figure S5 of the Supplementary Materials.

Although the above-mentioned combined effect of wind speed and wind direction to HC concentration is predictable, it is worth noticing the new VOC device responded in an expected way when it was interrogated under higher windy and cleaner air source conditions. This analysis future confirms the validity of the VOC device even in uncertain wind conditions.

## 4. Conclusions

In this paper, the new portable wireless QTF sensor-based VOC-sensing device system is presented. Comparison of the VOC device to its previous version indicates that improvements to make the device smaller, lighter, and more user-friendly have been accomplished. The device has demonstrated adequacy as a personal VOC exposure monitor. The different field tests to evaluate the analytical, functional and usability performances of the new VOC device and its sensor have been satisfactory. Results showed good correlations between: (1) The VOC device and commercial PID monitor in outdoor air with motor vehicle exhausts; (2) the VOC device and GC-MS to reassure negative detection of gas in an environment enriched with single gas of H_2_S; (3) good tolerance of the VOC device response to a barometric change, and (4) real-time correlation with CO. The VOC device also showed fast response towards changes in ambient environmental operational conditions such as wind and temperature inversion. In conclusion, this upgraded portable VOC device together with the pre-calibrated sensor cartridge and new user interface (application) can be used in different real-world scenarios and is applicable to personal daily use.

## Supplementary Materials

The Supplementary Materials are available online at http://www.mdpi.com/1424-8220/16/12/2060/s1.

## References

[1] Atkinson R., Arey J. (2003). Atmospheric degradation of volatile organic compounds. Chem. Rev..

[2] Brown K., Sim R., Abramson J., Gray N. (1994). Concentrations of Volatile Organic Compounds in indoor air—A review. Indoor Air.

[3] Win-Shwe T.-T., Fujimaki H., Arashidani K., Kunugita N. (2013). Indoor Volatile Organic Compounds and chemical sensitivity reactions. Clin. Dev. Immunol..

[4] Chagger H.K., Jones J.M., Pourkashanian M., Williams A., Owen A., Fynes G. (1999). Emission of volatile organic compounds from coal combustion. Fuel.

[5] Sofuoglu S. C., Aslan G., Inal F., Sofuoglu A. (2011). An assessment of indoor air concentrations and health risks of volatile organic compounds in three primary schools. Int. J. Hyg. Environ. Health.

[6] Lerchner J., Caspary D., Wolf G. (2000). Calorimetric detection of volatile organic compounds. Sens. Actuators B Chem..

[7] Martínez-Hipatl C., Muñoz-Aguirre S., Beltrán-Pérez G., Castillo-Mixcóatl J., Rivera-De la Rosa J. (2010). Detection of volatile organic compounds by an interferometric sensor. Sens. Actuators B Chem..

[8] Patel S.V., Mlsna T.E., Fruhberger B., Klaassen E., Cemalovic S., Baselt D.R. (2003). Chemicapacitive microsensors for volatile organic compound detection. Sens. Actuators B Chem..

[9] Smith P.A., Lepage C.J., Harrer K.L., Brochu P.J. (2007). Hand-held photoionization instruments for quantitative detection of sarin vapor and for rapid qualitative screening of contaminated objects. J. Occup. Environ. Hyg..

[10] Lingg R.D., Melton R.G., Kopfler F.C., Coleman W.E., Mitchell D.E. (1977). Quantitative Analysis of Volatile Organic Compounds by GC-MS. J. Am. Water Works Assoc..

[11] Rastrello F., Placidi P., Scorzoni A., Cozzani E., Messina M., Elmi I., Zampolli S., Cardinali G.C. (2013). Thermal Conductivity Detector for Gas Chromatography: Very Wide Gain Range Acquisition System and Experimental Measurements. IEEE Trans. Instrum. Meas..

[12] Deng C., Yang X., Li N., Huang Y., Zhang X. (2005). A Novel Miniaturized Flame Ionization Detector for Portable Gas Chromatography. J. Chromatogr. Sci..

[13] Cagan A., Schmidt H., Rodriguez J.E., Eiceman G.A. (2010). Fast gas chromatography-differential mobility spectrometry of explosives from TATP to Tetryl without gas atmosphere modifiers. Int. J. Ion Mobil. Spectrom..

[14] Su X., Dai C., Zhang J., O’Shea S.J. (2002). Quartz tuning fork biosensor. Biosens. Bioelectron..

[15] Kosterev A.A., Tittel F.K., Serebryakov D.V., Malinovsky A.L., Morozov I.V. (2005). Applications of quartz tuning forks in spectroscopic gas sensing. Rev. Sci. Instrum..

[16] Tsow F., Forzani E., Rai A., Wang R., Tsui R., Mastroianni S., Knobbe C., Gandolfi A.J., Tao N.J. (2009). A wearable and wireless sensor system for real-time monitoring of toxic environmental Volatile Organic Compounds. IEEE Sens. J..

[17] Wang R., Tsow F., Zhang X., Peng J.H., Forzani E.S., Chen Y., Crittenden J.C., Destaillats H., Tao N. (2009). Real-time ozone detection based on a microfabricated quartz crystal tuning fork sensor. Sensors.

[18] Negi I., Tsow F., Tanwar K., Zhang L., Iglesias R.A., Chen C., Rai A., Forzani E.S., Tao N. (2011). Novel monitor paradigm for real-time exposure assessment. J. Expo. Sci. Environ. Epidemiol..

[19] Deng Y., Chen C., Qin X., Xian X., Alford T.L., Choi H.W., Tsow F., Forzani E.S. (2015). Aging effect of a molecularly imprinted polymer on a quartz tuning fork sensor for detection of volatile organic compounds. Sens. Actuators B Chem..

[20] Chen C., Campbell K.D., Negi I., Iglesias R.A., Owens P., Tao N., Tsow F., Forzani E. (2012). A new sensor for the assessment of personal exposure to Volatile Organic Compounds. Atmos. Environ..

[21] Pitten F.A., Bremer J., Kramer A. (2000). Air contamination with volatile organic compounds (VOC's) and health complaints. Dtsch. Med. Wochenschr..

[22] Zheng H., Zhao X., Di J. (2009). Hydrogen sulfide removal from petroleum refinery by immobilized thiobacillus ferrooxidans in fixed-bed bioreactor. Pet. Sci. Technol..

[23] Klouda G.A., Connolly M.V. (1995). Radiocarbon (14C) measurements to quantify sources of atmospheric carbon monoxide in urban air. Atmos. Environ..

[24] Thomson G.W. (1946). The Antoine Equation for Vapor-pressure Data. Chem. Rev..

[25] Weisel C.P., Zhang J., Turpin B.J., Morandi M.T., Colome S., Stock T.H., Spektor D.M., Korn L., Winer A.M., Kwon J. (2005). Relationships of Indoor, Outdoor, and Personal Air (RIOPA). Part I. Collection methods and descriptive analyses. Res. Rep..

[26] Brown S.G., Frankel A., Hafner H.R. (2007). Source apportionment of VOCs in the Los Angeles area using positive matrix factorization. Atmos. Environ..

[27] Grober R.D., Acimovic J., Schuck J., Hessman D., Kindlemann P.J., Hespanha J., Morse A.S., Karrai K., Tiemann I., Manus S. (2000). Fundamental limits to force detection using quartz tuning forks. Rev. Sci. Instrum..

[28] Friedt J.-M., Carry É. (2007). Introduction to the quartz tuning fork. Am. J. Phys..

[29] Portland State Aerospace Society A Quick Derivation Relating Altitude to Air Pressure. http://www.psas.pdx.edu.

[30] Surridge A.D. (1986). Extrapolation of the nocturnal temperature inversion from ground-based measurements. Atmos. Environ..

