# Supplementary material for: A Novel Wireless Wearable Volatile Organic Compound (VOC) Monitoring Device with Disposable Sensors
Source: Sensors (Basel). 2016 Dec 3;16(12):2060. doi: 10.3390/s16122060 (PMC5191041; doi:10.3390/s16122060)
Supplement: Supplementary file 1 [file sensors-16-02060-s001.pdf]

Supplementary Materials: A Novel Wireless Wearable Volatile Organic Compound (VOC) Monitoring Device with Disposable Sensors

Yue Deng, Cheng Chen, Xiaojun Xian, Francis Tsow, Gaurav Verma, Rob McConnell, Scott Fruin, Nongjian Tao and Erica S. Forzani

1. Device and Sensor Specifications

Table S1 summarizes the specifications of the new VOC device with a MIP-modified TF sensor (MIP-TF sensor) for detection of hydrocarbons. The hydrocarbon sensor, as presented here, primarily detects hydrocarbon compounds, including aromatic, alkyl, and chlorinated hydrocarbons, with a measurement range from 2 to 320 ppm xylene equivalent concentrations. In addition, tests performed under different environmental conditions indicated that the device can be operated regularly under a temperature range of 32 °F to 113 °F (0 °C to 45 °C) and a humidity range of 0% to 95% RH (non-condensing). The accuracy of the sensor, when tested with artificial single hydrocarbon samples (e.g., xylenes), is better than 4% of the measured value.

The lifetime of the sensor is defined as the total amount of time sensors used (in hours) times the hydrocarbon concentration (ppmC) that the sensor has been exposed to, which is monitored by the device. As an example, the current device could be used for 465 h if it is being exposed to 1 ppmC VOC. The MIP-TF sensors are stored at −18 °C, which is a common temperature of a home freezer. Sensor shelf time will be discussed below.

Table S1. Device and Sensor Specifications.

Gas Detected	Hydrocarbons (HCs)
Operating temperature	32 °F to 113 °F (0 °C to 45 °C)
Operating humidity range	0 to 95% RH (non-condensing)
HCs Measurement range	2 ppm to 320 ppm
HCs fitting regression	$r^2 > 0.996$
HCs accuracy	±4% of measured value or better
Response time	Raw Data: 1 s per measurement<br>Calibrated concentration: 3 min per data point (adjustable)
Warm up time	<10 min
Lifetime	464 mC·h

2. Generation of Sensor QR Code

2.1. Langmuir Adsorption Isotherm

As described in main text and Figure 2, Langmuir adsorption isotherm is applied on calibration curve fitting. Langmuir adsorption could be expressed as [1]:

$$R = \frac{R_{\max} * \mathrm{c}}{\mathrm{K_D} + \mathrm{c}} \tag{1}$$

where R is the mass of adsorbed gas, which is proportional to the differential frequency change of QTF sensors, R_{max} is maximum amount of adsorbed gas that is represented by the maximum differential frequency change from the QTF sensors, and proportional to the maximum amount of analyte binding sites, c is the *o*-xylene gas concentration, and K_D is dissociation constant, which is defined as:

$$\mathrm{K_D} = \frac{[\mathrm{A}][\mathrm{B}]}{[\mathrm{C}]} \tag{2}$$

where [A], [B] and [C] are concentration of species [A], [B] and [C]respectively.

2.2. Temperature Correction

Due to the influence of temperature to chemical vapor pressure, the sensitivity varies under different temperature. Thus it is necessary to correct this effect on MIP-QTF sensor response [2].

As shown in Figure S1a, calibrations have been done on one MIP-QTF sensor under three different temperatures: 279 K, 298 K, and 312 K. Langmuir adsorption isotherm has been applied to all calibration curves.

Figure S1b,c summarizes the relationship between temperature with K_D and R_{max}, respectively. Results show that both K_D and R_{max} decrease as the temperature increases. Each sensor batch is calibrated at these three temperatures, which is considered as a unique characteristic and will be implemented into the batch QR code.

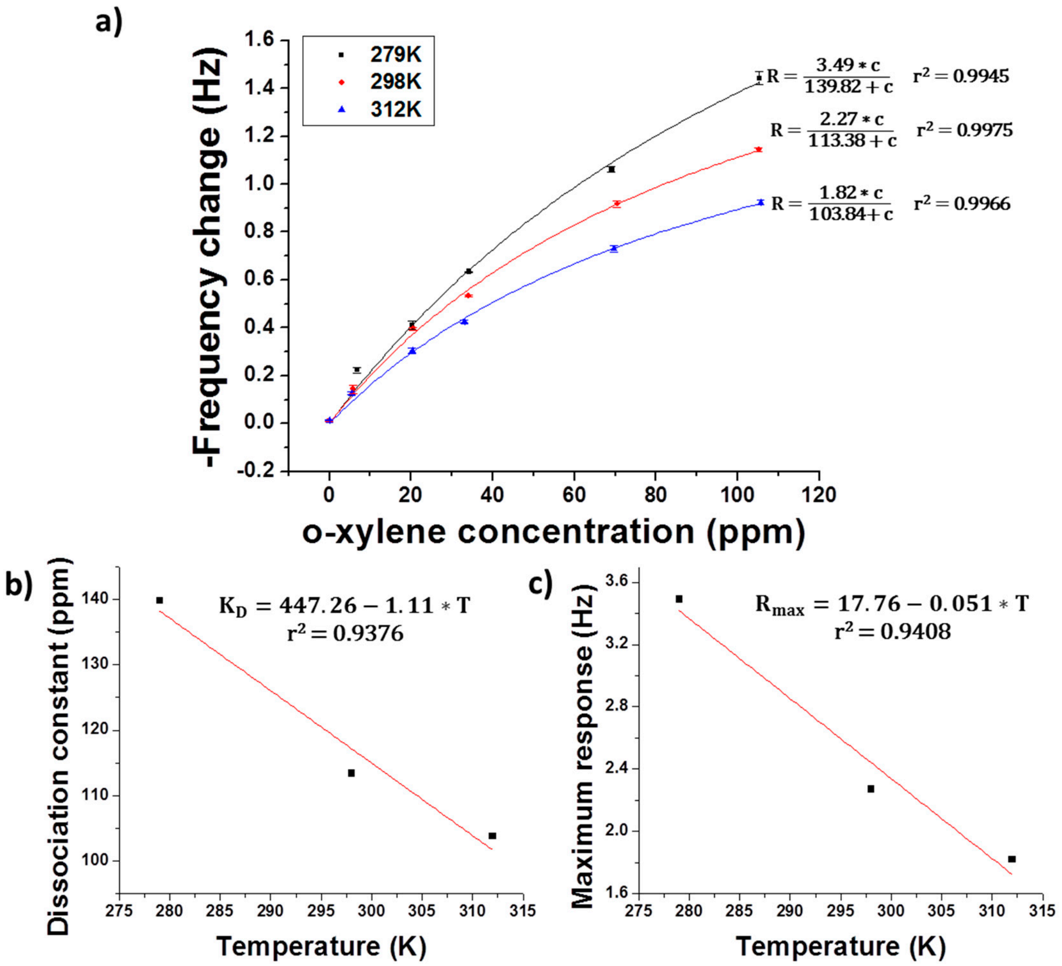

Figure S1. Temperature correction on sensing response, (**a**) Calibration under different temperature; (**b**) Linear fitting of K_D to temperature (T); (**c**) Linear fitting of R_{max} to temperature (T).

2.3. Sensor QR Code

There are two factors intrinsically influencing sensor response: time and temperature. As presented in previous publication [3], the sensitivity will decay over time. The decay pattern has been studied for one year, during which response of two batches of sensors to 40 ppm *o*-xylene was tested every other week or every month in the last three months (Figure S2). The patterns under different sample concentration are similar (not shown), thus we use 40 ppm *o*-xylene as a standard gas sample in this test.

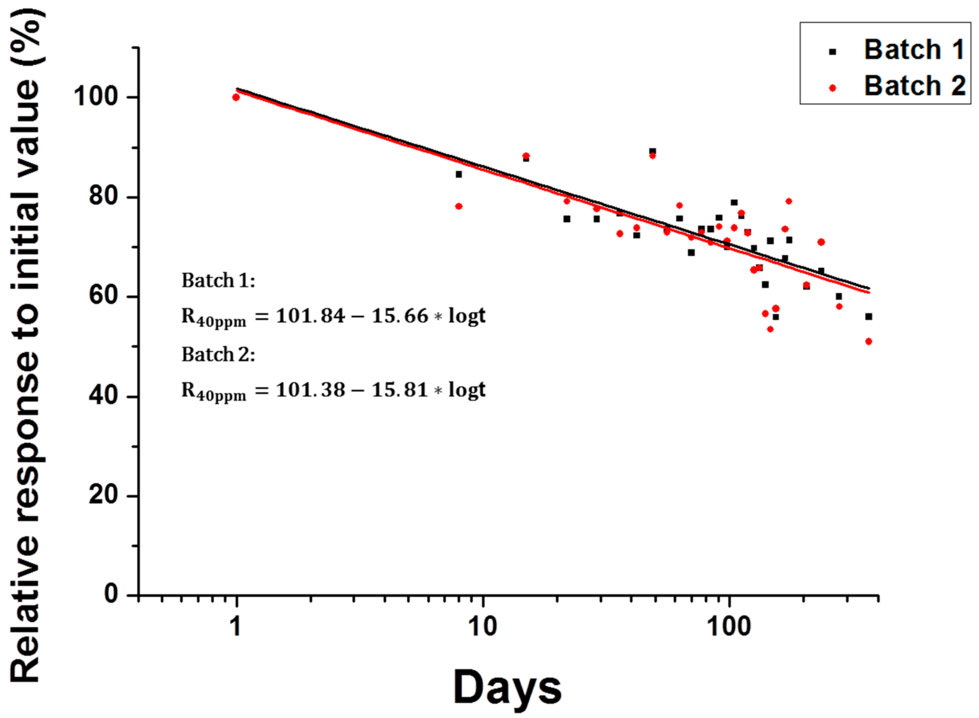

Figure S2. Sensor response degradation with time.

All information needed to correct the sensor response is summarized in Table S2. A 56 digits QR code is generated for each batch of sensor (typically with the amount of 200). In Table S2, *T* denotes temperature, *t* denotes time in dates and R_{40ppm} is the sensor response to 40 ppm pure *o*-xylene. With the pre-calibrated results and QR code, it is easier for users to get a precise monitor result without previous lab-calibration design requirement.

Table S2. Parameters used in QR code.

Parameter	Value	Comment
Variable A	−1.11	Slope of linear fitting of K_D to T
Variable B	447.26	Intercept of linear fitting of K_D to T
Variable C	−0.051	Slope of linear fitting of R_{max} to T
Variable D	17.26	Intercept of linear fitting of R_{max} to T
Variable E	−15.66	Slope of linear fitting of R_{40ppm} to log(t)
Variable F	101.84	Intercept of linear fitting of R_{40ppm} to log(t)
Manufacture Date	7 December 2014	Date when the manufacture is done
Batch number	201407150000000500	Packaging date & sensors number

3. Sensor Selectivity

Detailed synthesis and characterization of this molecularly imprinted polymer was presented before [3].The response of this MIP-QTF sensor to different gas analytes is shown in Figure S3. The polymer has good selectivity to the VOCs family, especially aromatic and hydrocarbon compounds. The non-response to carbon monoxide also demonstrates that VOC device result described in Section 3.2.4 is not due to the presence of carbon monoxide.

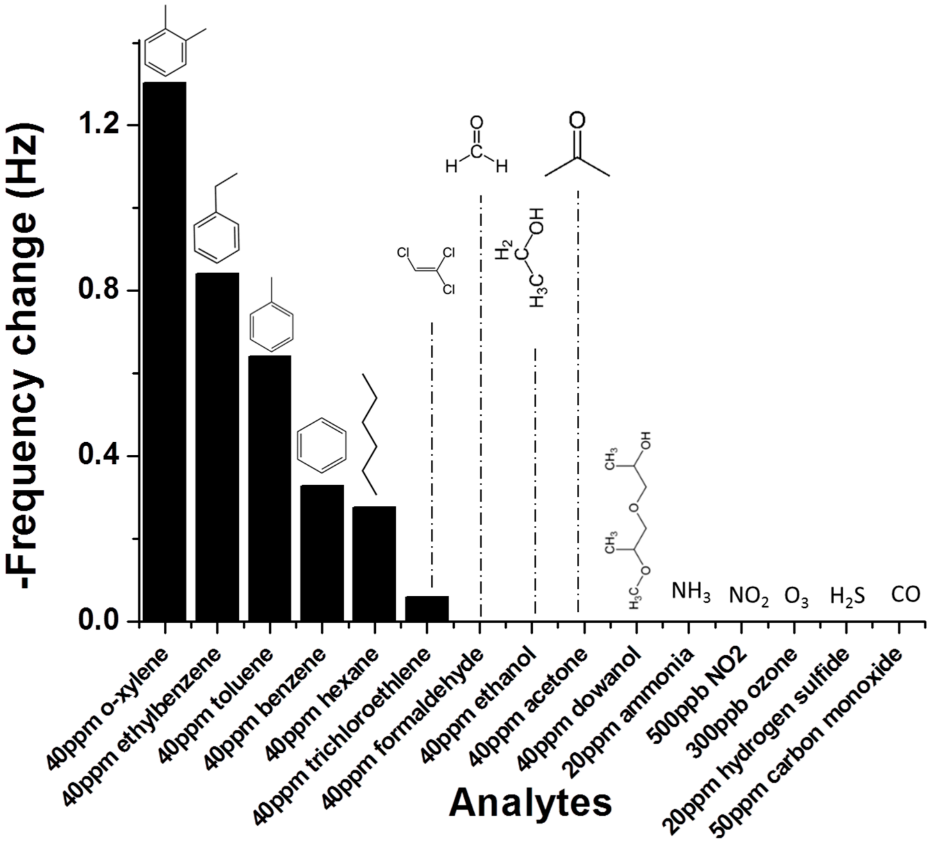

Figure S3. Response of MIP to different gas analytes.

4. Validation with Photo Ionization Detector (PID)

As described in the main text Section 3.2.1, the raw output from the VOC device and the PID in this field test qualitatively show that these two methods correlate well with each other in terms of general change over time. In order to quantitatively compare the results at same scale, the readouts are converted into relative responses (all data points are divided by the maximum value obtained from this test). The first three data points are excluded. A paired t test is performed on data shown in Figure S4.

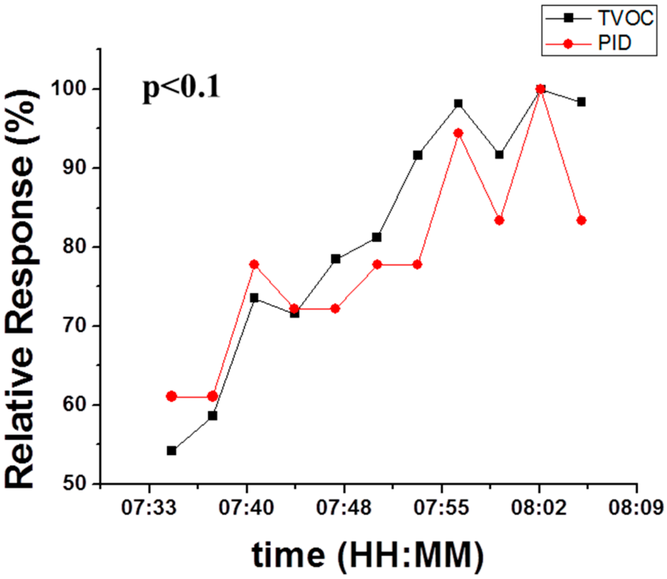

Figure S4. Relative response of the VOC device and Photo Ionization Detector for VOC levels assessed during a trip on Los Angeles Highway 101.

5. Real-Time Response of MIP-Modified Tf Sensors to Hydrocarbons

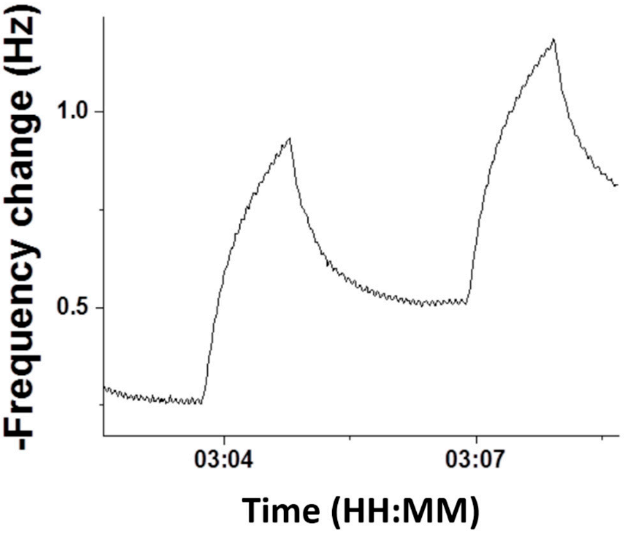

Figure S5. Real-time response of the VOC device to 40 ppm xylene on a modified tuning fork sensor. The baseline of the new injection is taken from the purging time slope assessed in the previous injection. Since the MIP has a high surface to volume ratio the number of binding sites are sufficient to conduct the measurements of the hydrocarbons during the lifetime of the sensors, which was defined to be 464 ppmC·h.

References

1. Goodrich, J.A.; Kugel, J.F. *Binding and Kinetics for Molecular Biologists*; CSHL Press: New York, NY, USA, 2006.
2. Deng, Y.; Chen, C.; Tsow, F.; Xian, X.; Forzani, E. Unraveling Fabrication and Calibration of Wearable Gas Monitor for use under Free-Living Conditions. In Proceedings of the 38th Annual International Conference of the IEEE Engineering in Medicine and Biology Society, Orlando, FL, USA, 16–18 August 2016.
3. Deng, Y.; Chen, C.; Qin, X.; Xian, X.; Alford, T.L.; Choi, H.W.; Tsow, F.; Forzani, E.S., Aging effect of a molecularly imprinted polymer on a quartz tuning fork sensor for detection of volatile organic compounds. *Sens. Actuators B Chem*. **2015**, *211*, 25–32.
